# Fruit and Vegetable Parenting Practices in Preschoolers: Initial Examination and Cultural Equivalency of a New Measure

**DOI:** 10.3390/nu18060974

**Published:** 2026-03-19

**Authors:** Lenka H. Shriver, Cheryl Buehler

**Affiliations:** 1 Department of Nutrition, University of North Carolina at Greensboro, P.O. Box 26170, Greensboro, NC 27402-6170, USA; 2 Human Development and Family Studies, University of North Carolina at Greensboro, P.O. Box 26170, Greensboro, NC 27402-6170, USA; cabuehle@uncg.edu

**Keywords:** fruit and vegetable consumption, parenting practices, preschool-aged children, measure development, measurement equivalency

## Abstract

**Background**: Encouraging fruit and vegetable (FV) consumption early in childhood is important for long-term healthy eating. Though parents play an important role in shaping children’s FV-related taste preferences and consumption, validated instruments assessing the range of parenting practices that specifically support young children’s FV intake are scarce. Furthermore, little attention has been given to low-income families, cultural inclusivity, and FV practices across different settings. The current study sought to conduct an initial examination and explore the measurement equivalency of a new FV parenting practices questionnaire (FVPPQ) across racially/ethnically diverse groups that address these gaps. **Methods**: Data for this paper came from a large project focused on parents’ FV parenting practices with young children enrolled in Head Start programs in the southern part of the U.S. Inclusion criteria were (a) parent/legal guardian being eighteen or older, (b) being the primary person responsible for child feeding, and (c) the child not requiring a special diet (e.g., diabetic). Using a multi-phases project approach, we (1) developed a preliminary conceptual map of parenting practice domains by reviewing existing measures on FV parenting practices; (2) completed and content-analyzed data from 18 focus groups (*n* = 62) to identify and further revise the preliminary conceptual map of domains, (3) administered a questionnaire with 11 domains of FV parenting practices, and then (4) empirically explored and reduced the measure while evaluating its content, construct, and criterion validity, and cultural equivalency across Non-Hispanic White, Hispanic White, and Black parents (*n* = 281). **Results**: Findings from Phases 1 and 2 generated a 107-item questionnaire that was reduced during phase 3 through a series of principal component and confirmatory factor analyses to the final FVPPQ with 21 items in four unique domains, showing good variability and inter-item consistency reliability: (1) Availability (5 items); (2) modeling (5 items); child-focused (5 items); and pressure (6 items). Three of the four domains evidenced cultural equivalency. **Conclusions**: The FVPPQ with four unique subscales demonstrated good content, construct validity, and partial measurement equivalency across racially/ethnically diverse groups of parents. Further confirmatory validation is warranted in larger samples, but the FVPPQ might be a promising and easily administered measure for research and applied interventions in nutrition, health behavior, and parenting contexts.

## 1. Introduction

Adequate fruit and vegetable (FV) consumption is essential for a wide range of health outcomes, including optimal nutritional status, reduced risk of chronic disease, and obesity prevention [1,2]. Despite these well-established benefits, FV intake remains sub-optimal across the U.S. pediatric population. National data indicate that only 7.1% of adolescents aged 12–19 meet the federal F intake recommendations and only 2% meet V recommendations [3,4]. Although some young children (aged 1–5 years) are not exposed to any FV on a daily basis, the intakes are significantly higher compared to adolescents, with approximately 68% and 51% of young children consuming F and V daily [5].

Early childhood represents a critical window for establishing healthy dietary patterns, including the regular consumption of various FV [6]. More importantly, it is a unique developmental period from a nutrition perspective because taste preferences develop rapidly during this time and the frequency and exposure to specific foods has a significant impact on the consumption of these foods in later years [7]. Thus, establishing adequate consumption of a variety of FV during early childhood influences not only children’s diet quality, but their health trajectories [4]. Furthermore, increasing FV consumption among young children is essential for broader societal and economic outcomes as it influences the success and cost effectiveness of major federally funded nutrition assistance programs, such as the National School Lunch program, as FV represent the largest yield of all food waste on school lunch trays in the U.S. [8,9].

Parents are the key gatekeepers of young children’s food intake by influencing which foods are available and by utilizing various practices to increase or limit the consumption of specific foods [10,11]. Unfortunately, FV represent foods that are harder to promote, especially vegetables (V), compared to fruit (F) and many other foods due to their natural bitter and/or tart taste as well as textures that young children find adverse [12,13]. As young children often experience different levels of food neophobia, sensory preferences, and other characteristics that influence their daily FV consumption, parenting practices specifically targeting FV are critical for the successful promotion of FV consumption among young children [12]. A systematic review and meta-analysis of 44 interventions [14] found several key strategies that promoted V intake among preschoolers, such as modeling, visual presentation, and repeated taste exposure. The authors found that children consumed more V when exposed to a specific V at least 8–10 times and when V were presented in their plain form rather than paired with dips or other flavors [14].

A number of measures of general parental strategies in relation to children’s overall food intake have been developed [15,16,17,18]. Yet validated instruments that focus specifically on parenting practices unique to FV promotion in early childhood are limited. Existing parental feeding-related measures often assess general constructs such as control, restriction, and pressure to eat, and many lack psychometric validation across diverse contexts and populations. A systematic review by Heller et al. [19] highlights gaps in feeding practice instruments for young children, particularly with respect to construct validity, reliability, and cultural applicability among low-income and racially/ethnically diverse families. Although previous attempts to examine effective and non-effective practices in relation to FV have provided valuable insights, these efforts have, so far, focused on V-related practices only, used mostly homogenous and middle-class samples, focused on school-aged children, and/or were generated by health/dietetic professionals rather than parents [20,21].

To date, the breadth of FV strategies utilized across racially/ethnically diverse groups of parents has not been well addressed in existing measures [20,21]. It is well established that FV-related behaviors and parental practices vary by cultural, socio-economic, and contextual factors. For example, previous research shows that preparation preferences and expectations for how V are served differs between low-income Hispanic and African American families, thus underscoring the need for culturally sensitive measurement tools [22,23]. Such tools must demonstrate construct validity, internal consistency, and measurement invariance across racial/ethnic groups to ensure the reliable interpretation of latent constructs underlying FV parenting practices and to ensure its generalizable use in research and practice.

The main purpose of this study was to conduct the initial investigations of a new measure of parenting practices for promoting FV consumption among racially/ethnically diverse, low-income preschool-aged children. Second, we aimed to explore its cultural relevance by assessing the measurement equivalency of the final set of the selected items across three racial/ethnic groups of parents. Using a multi-phase, mixed-method approach, we aimed to identify and test a large pool of items of parenting practices used for FV, and/or for F and V separately, in order to create a culturally equivalent instrument that captures the key dimensions of parenting practices relevant to young children’s F and V consumption.

## 2. Materials and Methods

### 2.1. Study Design and Sampling

Data for this multi-method study were collected as part of a project focused on parents’ FV parenting practices with their young children. The multi-study project consisted of 3 phases, and this paper focuses on the study conducted during Phase 3. The work done during Phases 1 and 2 is described here briefly because the selection of items for the 21-item measure of Fruit and Vegetable Parenting Practices finalized in the Phase 3 study was informed by the findings from the previous two project phases.

Phase 1 involved a review of the existing measures of FV parenting practices. Lists of items from existing measures of parenting practices were cataloged and a preliminary concept map of potential practice domains was created using methods described by Trochim and Linton [24]. This literature review of potential F and V feeding domains also resulted in a paper that provided practical advice for pediatricians [25].

Phase 2 of the project was a multi-method study that involved conducting focus groups with 62 parents (primary feeders) of preschool-aged children who were enrolled in one of five Head Start centers. Overall methodology and study procedures for our focus groups are described elsewhere [26]. A content analysis of the discussion guided our revision of the preliminary concept map of potential FV-practice domains [26] and elaborated the development of a comprehensive FV-related parenting practices questionnaire. A total of 18 focus groups were conducted with 29 self-identified Black participants (7 groups), 17 Hispanic participants (4 groups), and 16 Non-Hispanic White participants (7 groups). A majority of the focus groups ranged from 4 to 8 participants. The smaller sized focus groups among Non-Hispanic White parents was largely due low enrollment rates and scheduling difficulties within individual centers. In some cases, the focus group format was modified to an interview format in order to receive responses from all participants who attended the scheduled focus groups. Inclusion criteria were (a) being eighteen or older, (b) being the primary person responsible for child feeding, and (c) feeding a 3-to-5-year-old child who did not require a special diet (e.g., diabetic). This part of the project was approved as a stand-alone study by the University Institutional Review Board (protocol # 14-0119, 3 November 2014).

Nearly all participants were females (97%) and mothers (one grandmother). Distinguishing fruits from vegetables, the participants were asked to describe and discuss (a) strategies used and considered effective for encouraging FV consumption and (b) strategies considered not so effective. Following these discussions, mothers completed a preliminary 68-item questionnaire on FV parenting practices developed from the Phase 1 literature review and provided feedback to us regarding relevance, completeness, and clarity. Based on existing literature and measures, these 68 items covered eight conceptual domains of FV-specific parenting practices: Availability, accessibility, modeling, positive engagement, rules and routines, child involvement, pressure, and restriction.

Phase 3 was a correlational study of 281 parents of 3- to 5-year-old children (mostly mothers) recruited from 42 preschool sites in the southern U.S. Recruitment and data collection materials were available in English and Spanish. These participants differed from the 62 participants who were enrolled during Phase 2 (i.e., new recruitment was conducted). The survey was administered to participants by a trained interviewer and included six sections. One section consisted of the newly revised and elaborated FV-related parenting practices (107 items; listing available upon request) and two sections focused on the food frequency of the FV intake of the participant and of the child. The section on the FV-related parenting practices covered 11 conceptual domains of FV-related parenting practices: (1) Availability [11 items], (2) accessibility [12 items], (3) modeling [12 items], (4) verbal reasoning [7 items], (5) positive support [4 items], (6) reward [10 items], (7) monitoring [8 items], (8) practical techniques [9 items], (9) child inclusion [10 items], (10) pressure [10 items], and (11) restriction [14 items]. Participants were given $25 for their involvement in Phase 3. The cross-sectional part of the project (Phase 3) was approved as a stand-alone study by the University Institutional Review Board (#15-0072, 18 February 2015).

### 2.2. Participant Characteristics and Data Collection Procedures

Participant eligibility and characteristics. Parents or legal guardians aged 18 or older with a child enrolled in one of the participating Head Start programs and who were the primary feeder for the child were eligible for study inclusion. Participants also needed to self-identify as Black, Hispanic White, or non-Hispanic White, and be parenting a child who did not have any medical conditions that required a special diet (e.g., diabetes).

The average age of participants was 31.9 (*SD* = 7.8) and 94% were female (see Table 1). About 38% of the participants identified as Black (*n* = 106), 36% identified as Hispanic White (*n* = 100), and 27% (*n* = 75) identified as White. Within the limits imposed by the eligibility criteria, the sample was diverse in terms of racial/ethnic identification and educational attainment. Sixty percent lived with a partner. Using the Body Mass Index (BMI) classification criteria from the Center for Disease Control [27] and participants’ self-reported weight and height, 24% were categorized into a healthy weight category, 29% were in the overweight category, and 43% were in the obese category. On average, participating children were 4.35 years old (SD = 0.70), and the sample was evenly split across males and females (52% male children). Child BMI-for-age percentile averaged 75.6 (SD = 28.2), with 38% of children being classified as obese and 9% as overweight.

Data collection procedures. After establishing study eligibility, individual interviews that included completing questionnaires and the interviewer-administered food frequency assessment were scheduled at the participant’s convenience and preferred location (e.g., Head Start centers, our research lab). Childcare and transportation services were provided upon request. Adult participants provided written informed consent and signed permission forms for the release of child birthdate and anthropomorphic information from childcare records.

A six-part questionnaire was completed first during the interview. English and Spanish versions of the questionnaires were available. The Spanish version was created by an experienced Hispanic researcher using forward and backward translation techniques. Importantly, given the central focus of this study, this researcher also provided an assessment of the cultural relevance of the measures and data collection procedures. Interviewers were available to provide needed assistance for the questionnaire completion, as well as for conducting the food frequency assessment. Household aids such as cups, tablespoons, and plates were available to assist participants with reporting the FV intake of their child and themselves as accurately as possible. Data collection for participants whose primary language was Spanish was done by a fluent, trained Spanish-speaking researcher.

### 2.3. Study Measures and Variables

Fruit and Vegetable Parenting Practices questionnaire (FVPPQ). Following Phase 2 of the overall project, the 107-item questionnaire that consisted conceptually of 11 domains of FV parenting practices was reduced to a 21-item measure that conceptually and empirically captured four domains (Table S1). The process of reducing and grouping items is described below in Section 3. Most questionnaire items begin with the phrase “During a typical week, how often do you…” The 4-point response format was never (0), sometimes (1), often (2), and almost always (3). Inter-item consistency for the four subscales and the total measure is good with all Cronbach alpha estimates greater than 0.80 (Table 2). The subscales and total score have good variability and are not skewed nor kurtotic.

Measures/variables used to assess content validity of the FVPPQ. Content validity was assessed by scrutinizing items as they reflect the concept maps created during the first and second phases of the project. For this current study in Phase 3, content validity also was assessed by correlating the FVPP with established measures of parents’ feeding practices to identify potential areas of content similarity and difference. The four FVPP subscales were correlated with three subscales from the Child Feeding Practices (CFQ): Monitoring, restriction, and pressure to eat [28]. The four FVPP subscales also were correlated with the demandingness and responsiveness subscales from the Caregivers’ Feeding Style Questionnaire [15]. An additional assessment of content validity was conducted using correlations with the Fruit, Juice, and Vegetable Availability Questionnaire [29].

Measurement equivalence across Hispanic White, Non-Hispanic White and Black parents also was examined. The cultural relevance and appropriateness of using the FVPPQ with parents who identify with each of these racial-ethnic groups was assessed by conducting a series of confirmatory factor analyses across the three groups of parents. This assessment provided evidence used to evaluate (a) content validity from a perspective of cultural relevance and configural invariance [same items in a subscale for each group], (b) metric [equivalent factor loadings across the three groups] and scalar equivalence [equivalent item intercepts across the three groups], and (c) adequate inter-item consistency reliability of the FVPPQ subscales for each group of parents.

Measures/variables used to assess criterion-related validity of the FVPPQ. Criterion-related validity was assessed by correlating the FVPPQ subscales with child weight status and children’s consumption of fruit and vegetables. Child weight status was measured as BMI-for-age percentile (Epi Info, CDC, version 2007). It also was measured using the parent concern regarding child weight subscale from the CFQ. Children’s consumption of 21 fruits and 28 vegetables during the past week was assessed using a modified version of the Slu4Kids Food Frequency Questionnaire (FFQ) [30].

Measures/variables used to assess construct validity of the FVPPQ. Four variables were expected to be associated with FVPPQ variables: Parent and child preferences for a variety of fruits and for a variety of vegetables. Participants were asked to report on their own taste preferences for 21 different fruits and 28 vegetables as part of the FFQ [30,31]. They also reported on the child’s taste preferences with regard to these fruits and vegetables using the same measure that was part of the FFQ measure [31]. The response format for each listed FV was: Never had it (0), hate it (1), does not like it (2), like it (3), and love it/my favorite (4).

### 2.4. Statistical Analyses

Descriptive statistics, inter-item consistency (i.e., Cronbach’s alpha), and zero-order correlations were procured using the Statistical Package for Social Sciences (IBM SPSS Statistics, version 29, IBM Corp., Armonk, NY, USA, 2023). Principal component analysis (PCA) was utilized for initial analyses given this study was focused on item reduction and the identification of useful central patterns of item covariance. In applied psychometrics, the use of exploratory PCA is considered an appropriate first step toward measure/scale development because it informs subsequent confirmatory modeling [32,33]. In these PCA analyses, loadings greater than 0.39 were considered useful for continued inclusion considerations and we also expected that the primary and secondary loadings should differ by more than 0.20 to help ensure adequate discriminant validity. In addition, we looked for similar patterns across the results based on orthogonal and oblique factor/component rotations.

Given this sample size of 281 was not adequate to include all 107 items in one PCA, we conducted these analyses in a series of nine PCAs that used parcels of 15 randomly selected items. Using random subsets of items in a series of PCAs is an appropriate item-reduction procedure when there is a limited sample size in relation to a large number of items which can produce unstable solutions [34]. As noted and justified by Velicer and Fava [35], using a random selection of smaller sets of items for these analyses is similar in logic to random sampling procedures that are utilized to avoid “overfitting to idiosyncratic sample variance while identifying stable covariance structures across subsets”.

Confirmatory factor analyses were conducted using Mplus (version 8.6, Los Angeles, CA, USA) [36] to provide model fit information for the total sample and to conduct the cultural relevance evaluation across the three racial/ethnic groups. Three fit indices are reported: Chi-square, SRMR, and the CFI for the single-factor models. The change in chi-square also was reported when comparing measurement equivalence across groups. Statistical significance was set at *p* < 0.05 for all analyses.

## 3. Results

### 3.1. Fruit and Vegetable Parenting Practice Questionnaire (FVPPQ)

The original 107-item FFPVQ was reduced to a 21-item measure in a series of mixed method procedures. This item-reduction process that included several quantitative and qualitative procedures was consistent with the grounded, multiple-method approach adopted for the larger project that involved the continual consideration and integration of existing literature and new findings.

As a first step, PCAs were conducted and findings were scrutinized using information garnered in the extensive literature review (Phase 1 of the larger project) and the information garnered from the focus groups (Phase 2). A total of nine PCAs were conducted with the goal of identifying common underlying groups of items across the multiple items. We ceased this part of the item-reduction process after 9 PCAs because similar patterns of findings had emerged. In addition to identifying common patterns across analyses, the results from each analysis were compared with the preliminary concept map of FV parenting practices developed from existing literature in Phase 1 and with the qualitative and quantitative information gleaned from the Phase 2 focus groups. An additional consideration in the process of final item reduction was the need to retain a mix of items referring to F only, V only, and both FV in the statement. One of the limitations of the existing research on parenting practices and children’s FV consumption is that F and V are examined together as one outcome while parents likely use different strategies when encouraging their child’s consumption of F versus V. Based on our extensive review of the existing literature and the analysis of our own focus group data, we chose to include some items specifically asking only about F or V, but not both so participants could think about their unique F- and/or V-related strategies when responding to the statements.

This process resulted in the selection of 21 items that represented four conceptual domains of FVPPQ: Availability (5 items), modeling (5 items), child-focused (5 items), and pressure (6 items). The selected items are listed in Table 3. As shown in Table 2, the subscales and total measure had good variability and inter-item consistency reliability.

The selection of these 21 items using PCA, along with considerations generated from information garnered during Phases 1 and 2 of the larger project, was followed by a PCA conducted with the total sample using all 21 items. This PCA yielded four components with eigenvalues greater than 1 and explained 60.60% of the variance. Each item had high primary loadings on the expected dimension (Table 3). Each of the items also had at least 0.20 between the primary and secondary loadings, providing some evidence of discriminant validity. The orthogonal and oblique rotations had similar patterns of findings, and a sample size of 281 was adequate for this type of analysis [37]. The results shown in Table 3 are from the PCA with a varimax orthogonal rotation. The availability subscale was correlated 0.65 (*p* < 0.001) with modeling and 0.65 (*p* < 0.001) with -child-focused. Modeling was correlated 0.67 (*p* < 0.001) with child-focused. The pressure subscale was correlated 0.05 (*p* = 0.44) with availability, 0.05 (*p* = 0.45) with modeling, and −0.02 (*p* = 0.80) with child focused. Given that the pressure domain of parenting practices has had mixed results with expected child health outcomes in previous research [38], it is important to note that the inter-item correlations of the pressure items with the other items in the 21-item measure were small (e.g., 0.04, −0.05) and that pressure was associated positively with the FVPPQ total score (*r* = 0.41, *p* < 0.001). Given these findings, the 21-item measure with four FV parenting domains was retained.

Finally, the primary goal of the current study was to create a measure with cultural relevance. To this end, we first conducted confirmatory factor analyses for each dimension within each racial-ethnic group. The central purpose of this procedure was to obtain estimates of model fit for each group of parents. Twelve confirmatory factor analyses (CFA) were conducted to estimate model fit for each dimension within each racial-ethnic group (Table 4). WLSMV was used for CFA estimation given the items are ordinal level of measure [39]. Each of the four subscales demonstrated a good model fit with CFIs greater than 0.96 and SRMR values less than 0.09 [40]. These findings provided preliminary evidence of configural measure equivalence across Black, Hispanic White, and Non-Hispanic White parents given they demonstrated that the subscale items were relevant for each of the proposed domains of FV feeding practices. Inter-item consistency reliability also was good for all subscales with the exception of the availability subscale for Hispanic White parent (lower but adequate α = 0.65).

Measurement equivalence analyses across Black, Hispanic White, and Non-Hispanic White parents were conducted on the FVPPQ subscales (Table 5). Each of the four subscales demonstrated that the factor loadings were equivalent across racial-ethnic groups (i.e., metric equivalence). Three of the four subscales also demonstrated that the thresholds were equivalent across racial-ethnic groups (i.e., scalar equivalence): Pressure, availability, and modeling.

The child-focused subscale had thresholds that varied across two of the groups. Follow-up analyses indicated that three of the five items differed for Hispanic White and non-Hispanic White parents. Hispanic White parents had greater thresholds for the items focused on having prepared vegetables within the child’s reach and fruits ready and washed. Non-Hispanic White parents had a higher threshold for the item that focused upon including the child in FV preparation.

### 3.2. Correlations Between Relevant Variables and the FVPPQ Subscales and Total Score

Additional evidence of content validity was garnered by correlating the FVPPQ subscales and the total summary score with (a) the monitoring, restriction, and pressure-to-eat subscales from the CFQ, (b) the demandingness and responsiveness subscales from the CFSQ, and (c) the fruit and vegetable availability subscales from the Fruit, Juice, and Vegetable Availability Questionnaire (Table S2). There was almost complete data on the 21 items in the FVPPQ (less than 0.002 missing values), and the subscale and total summary scores were computed by creating a mean score. A mean score was computed rather than a summed score, given the number of items included in the summary scores varied. As such, the values on the FVPPQ summary scores ranged from 0 (rarely) to 3 (almost always).

The FVPPQ pressure subscale was associated positively and significantly with CFQ’s restriction, pressure to eat, and in particular, CFSQ’s demandingness (*r* = 0.51, *p* < 0.001). It was associated negatively with responsiveness in the CFSQ (*r* = −0.37, *p* < 0.001). The FVPPQ availability subscale was associated positively with CFSQ’s responsiveness and vegetable availability. The FVPPQ modeling and child-focused subscales were also each associated positively with CFSQ’s responsiveness and FV availability.

Evidence of criterion-related validity was examined by correlating the FVPPQ subscales with child fruit intake during the past 7 days, child vegetable intake, and child BMI-for-age percentile (Table S2). The total FVPPQ measure was significantly associated with child FV intake (*r* = 0.28, *p* < 0.001 for both F and V). Except for the association between pressure and child fruit intake, all of the FVPPQ subscales were associated significantly with child fruit and vegetable intake. Statistically significant correlations ranged from −0.14 (FVPPQ pressure with child vegetable intake) to 0.34 (FVPPQ child-focused practices with both child fruit and vegetable intake). None of the FVPPQ subscale scores were associated with child BMI-for-age percentile.

Evidence of construct validity was garnered by correlating the FVPPQ subscales with parents’ and children’s preferences for fruits and vegetables (four variables) (Table S2). Availability, modeling, child-focused, and the total score were associated positively with parents’ preferences for both fruits and vegetables and children’s preferences for vegetables. The statistically significant correlations ranged from 0.12 (total score and child vegetable preferences) to 0.20 (availability with parent and child vegetable preferences).

Correlations also were estimated with household income, maternal education, and maternal age. None of the associations were statistically significant with the FVPPQ total score or any of the subscale scores. Correlations ranged from 0.00 (FV availability and education) to −0.12 (pressure and parental age).

In summary, the new 21-item FVPPQ included four unique domains: Availability, modeling, child-focused, and pressure. The measure demonstrated extensive evidence of content validity. There was also evidence of criterion and construct validity. The measure was metric equivalent (i.e., factor loadings) and scalar equivalent (i.e., thresholds) across Black, Hispanic White, and Non-Hispanic White parents, with minor exceptions of a few differences in item intercepts between Hispanic White, and Non-Hispanic White parents.

## 4. Discussion

The current study presents our conceptualization and initial validity investigations of a new measure of parenting practices targeting F and V among 3–5-year-old children. Furthermore, we present findings related to measurement equivalency across Black, Hispanic White, and Non-Hispanic White parents, providing preliminary evidence that the new measure is suitable for utilization across racially/ethnically diverse families with preschool-aged children. We demonstrate that the 21-item FVPPQ has good validity and inter-item consistency reliability and represents an appropriate tool for assessing FV-related parenting practices in racially/ethnically, as well as socio-economically diverse samples of parents of 3–5-year-old children.

Three of the four unique parenting practice domains in the FVPPQ (i.e., availability, modeling, and child-focused) align closely with the more global, second-order construct of parental responsiveness [15,41,42]. The correlations among these three practice domains were 0.65 and 0.67, indicating about 45% shared variance. Responsive parents are characterized by both high responsiveness and high demandingness while the focus of their strategies is the child rather than the parent and/or the parental control [15,41]. Items representing these three domains have been associated with higher FV consumption among children in previous research and thus are considered to be positive in nature from the parenting perspective [42,43,44]. It is important to note that while studies have identified FV availability as a key factor associated with children’s higher FV consumption, our findings indicate that additional strategies, such as parental FV modeling (and thus indirectly parental taste preferences for FV) and having the child actively engaged in planning, choosing and preparing FV, are critical for promoting FV consumption among young children [42]. Although the results from this study support the contention that a higher-order responsiveness construct exists for FV feeding practices with young children, we believe that it is premature to aggregate these FV-related feeding practices into a single responsiveness score. Retaining three separate availability, modeling, and child-focused scores will facilitate the future examinations of important questions focused on young children’s FV preferences, FV consumption, and health indicators, supporting the conclusion that collapsing practice domains into a higher-order construct at this point in the measure development is premature.

The FVPPQ also includes the pressure subscale. The items in this domain were reverse coded in the total score as the use of parental pressure generally indicates an undesirable approach to child feeding. Pressure aligns with the more global parenting construct of high demandingness with low responsiveness and has been linked, in some (though not all) studies, to adverse child outcomes, such as higher obesity risk, lower taste preferences for FV, and higher consumption of high-sugar and/or high-fat foods [44,45]. We have found that pressuring behaviors are grounded in the daily experiences of many parents. In fact, a variety of pressuring practices were heavily reported by parents in our focus groups across racial/ethnic groups, and similarly a wide range of pressure feeding strategies have been highlighted in the existing literature and in previously developed measures of parental feeding practices [20,21]. Our findings also support previous research in that most parents tend use a mixture of positive and negative strategies on a daily basis, with some being more responsive and child-focused while some might fall into the domain of pressure [20]. Our work here is significant because it contributes a new, brief measure focused on FV that captures both responsive strategies and also some of the most commonly utilized non-responsive parenting practices that constitute pressure.

Our analytical approach in the current study was unique due to our focus on developing a measure of FV parenting practices that are culturally relevant for Black, Hispanic White, and Non-Hispanic White parents of young children. We also aimed to achieve applicability across socioeconomic circumstances, following a call by Heller et al. [19] for new measures that demonstrate not only adequate validity and inter-item consistency reliability, but also cultural applicability. The 21 items selected for the FVPPQ met these goals, with several results supporting this conclusion. English and Spanish versions of the questionnaires were available to the study participants. The original 107-item version of the measure was reviewed and revised accordingly by a Spanish-speaking professional with extensive experience in Head Start programs. The same research assistant also created the Spanish version of the questionnaire using forward and backward translation techniques. Importantly, given the central focus of this study, this research assistant also provided an assessment of cultural relevance of the measures and data collection procedures. Analyses were conducted within each racial/ethnic group to ensure that each of the items selected for the final 21-item version of the measure were relevant, providing additional evidence of good content validity. The final version also was examined for measurement equivalence across racial/ethnic groups of parents. These analyses demonstrated that each of the items contributed to a given domain across racial/ethnic groups (i.e., factor loading equivalent), and that most of the items had the same amount of measurement error across groups (i.e., scalar equivalent). Importantly, the subscale scores and the total FVPPQ score were not associated with parents’ educational attainment, age, or household income. As such, the FVPPQ measure is applicable across diverse samples of parents of young children.

In addition to focusing specifically on feeding practices related to FV, a major focus of this work was to develop a measure that included some items that distinguish parenting practices utilized for feeding F from those for feeding V. Existing measures of feeding practices often have combined F and V in the same parenting practice statement, thus not distinguishing between practices that work for F but may not work for V. This distinction was important given that V are rejected more often by young children than fruit due to their bitter taste [26,46]. After extensive validity processes used to reduce the measure from 107 items to 21 items, nine items in the FVPPQ ask only about V, six items ask only about F, and six items ask about both F and V in the same statement. V-focused items tended to fit as part of the pressure feeding domain, whereas F-focused items tended to fit as part of the child-focused feeding domain. This pattern would not have been revealed if all of the items referred to both fruits and vegetables and deserves additional attention in future research. The evidence presented here of strong content validity supports the contention that this constellation of items was a good approach to measure construction and reduction and replicates a theme that emerged during the focus group regarding the importance of distinguishing fruit and vegetable feeding practices [26].

In addition to cultural applicability and a distinction between fruit and vegetable feeding practices with young children, our initial 107-item measure included items that addressed a variety of settings. We included items that focused on weekday and weekend practices, eating at home and away from home, and meals and snacks. This intention was focused on enhancing the content validity of the final measure. The 21-item FVPPQ measure includes nine items that specify snack or meal or at home versus eating away from home. The remaining 12 items asked about a given practice during a typical week. All of these items demonstrated racial/ethnic equivalency.

The FVPPQ total score and subscale scores demonstrated criterion-related validity when considering the criteria of child FV intake but not child BMI-for-age percentile. Both child fruit intake, and importantly vegetable intake, were associated with FV availability, modeling, and being child-focused when feeding FV, as well as the total score. This corresponds with previous research on responsive feeding styles [41,42]. Neither the total FVPPQ score nor the subscales, however, were associated with children’s BMI-for-age percentile in our sample. This finding is not surprising given the cross-sectional nature of our data and the fact that children’s weight is influenced by many other factors besides FV consumption, such as physical activity [47,48]. In addition, FV do play an important role in diet quality, but FV intake has been moderately associated with lower obesity among adults only, not children [2]. Further research is warranted to examine longitudinal associations between parental practices related to children’s FV consumption, actual FV intake, and weight outcomes across childhood.

It is well established that parents’ and children’s preferences for fruits and vegetables are strongly associated with children’s FV intake [49]. In our study, parents’ taste preferences for FV, measured separately, were each associated positively with the FVPPQ total score, as well as the availability, modeling, and child-focused practice domains. These associations provide some evidence of construct validity for our new FVPPQ measure, in addition to affirming the importance of parents’ FV preferences for shaping shopping and serving FV-related behaviors. Children’s preferences were also associated with the FVPPQ scores, but only their taste preferences for vegetables. In addition to significant associations with the subscale scores, the pressure subscale was related to children’s lower preferences for vegetables. This association could be explained by the fact that some children are naturally more sensitive to the bitter and/or tart taste of vegetables than others, thus refusing vegetables more strongly than what is developmentally typical for young children [46,50]. Consequently, parents of such taste-sensitive children might resort to applying a greater degree of pressure than other parents whose children are less sensitive to these tastes.

The current study has several strengths. First, this is the first measure of FV-related parenting practices that has been examined for use across racially/ethnically diverse samples of parents of young children. Second, we utilized a multi-phase approach that included a comprehensive review of literature and existing measures of parenting feeding practices and analyses of original focus group and survey data to generate a large pool of parenting practices used to encourage FV consumption among 3–5-year-old children. Third, we utilized data from parents with diverse ethnic/racial, educational, and income backgrounds. Lastly, we were able to empirically reduce the original strategies to a 21-item measure that is brief and easily administered in a variety of settings, such as childcare programs, pediatrician offices, and healthy eating programs.

There also are several study limitations. First, we recruited our participants from a convenience sample of Head Start centers who agreed to be part of our study; thus our findings do not represent all families enrolled in Head Start program in NC. Also, it is possible that parents who volunteered for the study were more interested in nutrition and/or struggled more with offering FV to their children compared to other parents. Future studies that use the FVPPQ also should assess parents’ interests in healthy eating as well as child feeding difficulties in order to begin assessing selection effects that often accompany community-based prevention and intervention programs. Second, our findings are not representative of ethnic/racial groups other than Black, Hispanic White and Non-Hispanic White. Third, not all focus groups conducted in Phase 1 had ideal size; however, these challenges have been reported and well documented in previous studies that are community-based and recruit participants from socio-economically disadvantaged populations. Fourth, temporal stability of the FVPPQ could not be established within this study due to limited funding and feasibility; thus, test–retest reliability should be established in future research with a larger sample. Fifth, the criterion validity testing was limited to using child BMI-for-age percentile as other indicators suitable for criterion validity testing were not available. In addition, the child-focused subscale demonstrated some aspects of measure equivalence across ethnic/racial groups of parents but evidenced some differences between Hispanic White and non-Hispanic White groups with regard to scalar equivalence. As such, future research that compares child-focused FV parenting practices across these two groups needs to interpret any found mean differences within the context of possible differences in item difficulty or bias across groups. Finally, the use of parcels and PCAs is just a first step in identifying FV parenting domains. Future research needs to replicate this work in independent samples of parents with young children.

## 5. Conclusions

The 21-item FVPPQ is a new tool that can be administered to parents of young children without excessive burden and as part of a variety of health, nutrition, and/or parenting programs. Our study established initial evidence of content, criterion, and construct validity of the new measure, as well as its measurement equivalence across Non-Hispanic White, Hispanic White, and Black parents. Future research that will utilize the FVPPQ is warranted to evaluate FV parenting practices across large samples and to examine which parental behaviors and/or domains are associated with the most optimal FV-related outcomes among young children. The FVPPQ can also be utilized as part of future intervention work that is focused on optimizing FV consumption in racially/ethnically and socio-economically diverse families with young children. Finally, longitudinal research that will assess children’s as well as parents’ FV intake and child weight outcomes and expand the use of this new measure beyond the racial/ethnic groups represented in the current study, is warranted.

## Figures and Tables

**Table 1:** Parent and Family/Household Descriptives (*N* = 281).

Characteristics/Variables ^1^	M (SD)/%
Maternal Age, years	31.9 (7.8)
Maternal Body Mass Index	30.3 (7.4)
Weight Status ^2^	
Underweight	4 (1%)
Healthy Weight	67 (24%)
Overweight	80 (29%)
Obese	120 (43%)
Household Income	
Less than $10,000	93 (33%)
$10,000–34,999	163 (58%)
More than $35,000	21 (7%)
Race/Ethnicity ^3^	
Non-Hispanic White	75 (27%)
Non-Hispanic Black	106 (38%)
Hispanic/Other/Multiracial	100 (36%)
Educational attainment	
≤High school diploma/GED	153 (54%)
Some college	83 (30%)
2-year/4-year college degree	33 (12%)
Post graduate work/degree	6 (2%)
Other	6 (2%)

^1^ Some variables are based on fewer than 281 participants as some had missing data at random; ^2^ Classified based on Body Mass Index (weight in kg/height in m^2^) and the cut off values developed by the Center for Disease Control and Prevention [27]; ^3^ Self-identified data collected from participants.

**Table 2:** Descriptive Statistics of the Fruit and Vegetable Parenting Practice Questionnaire (FVPPQ).

Measure/Subscale	Mean (SD)	Range	Skewness	Kurtosis	Cronbach’s Alpha
FVPPQ					
Pressure	0.88 (0.67)	0–2.67	0.58	−0.44	0.83
Availability	2.15 (0.64)	0–3	−0.59	−0.04	0.81
Modeling	1.71 (0.82)	0–3	−0.10	−0.88	0.87
Child-Focused	1.68 (0.75)	0–3	−0.18	−0.55	0.82
Total (21 items)	1.57 (0.50)	0.05–2.76	−0.33	0.15	0.88

**Table 3:** Principal Component Analysis of the FVPPQ ^1^ 21 Items (Varimax Orthogonal Rotation).

FVPPQ Item	Pressure	Availability	Modeling	Child-Focused
During a typical week, how often do you tell/ask this child to eat at least half of their vegetables so that s/he can be done eating?	**0.78**	0.06	0.02	0.03
How often do you ask/tell this child to take a few more bites of a vegetable?	**0.78**	0.14	0.05	−0.14
During a typical week, how often do you tell your child there is no dessert if they do not eat their vegetables?	**0.75**	0.12	−0.13	0.19
During a typical week, how often do you barter with this child to eat their vegetables?	**0.72**	−0.12	0.06	−0.16
During a typical week, how often do you beg your child to eat his or her fruit or vegetables?	**0.69**	0.00	0.03	−0.21
During a typical week, how often do you warn your child you will take something away (toy, playtime) if she or he does not eat their fruit or vegetable.	**0.68**	−0.14	0.03	0.28
How often do you buy fruit or vegetables when shopping for food?	0.01	**0.72**	0.23	0.18
During a typical week, how often do you include at least one fruit or vegetable that your child likes in meals or snacks?	−0.002	**0.70**	0.31	0.19
How often do you provide vegetables for this child in the form s/he prefers—like frozen if s/he likes frozen, raw if that is the preference, or cooked if that is preferred?	0.03	**0.70**	0.04	0.21
How often are vegetables in your home during a typical week (fresh, canned, jarred, frozen, or 100% vegetable juice)? Please do not think about fried vegetables like French fries.	0.00	**0.68**	0.24	0.16
During a typical week, how often do you include/serve vegetables in this child’s meals served at home (e.g., lunch, dinner).	0.03	**0.63**	0.27	0.38
During a typical week, how often do you eat a fruit in front of your child when eating out (i.e., restaurant, fast food, buffet)?	−0.03	0.07	**0.82**	0.09
During a typical week, how often do you eat fruit as dessert in front of this child?	0.06	0.30	**0.71**	0.25
During a typical week, how often do you eat a vegetable for a snack when your child can see this?	0.07	0.34	**0.68**	0.29
During a typical week, how often do you eat fruit in front of this child at meals?	−0.03	0.23	**0.66**	0.43
During a typical week, how often do you eat fruit for a snack when your child can see this?	0.06	0.37	**0.66**	0.33
During a typical week, how often do you have vegetable pieces where your child can easily reach them?	−0.05	0.16	0.20	**0.80**
During a typical week, how often do you have fruits ready to eat by peeling, sectioning, or cutting into small pieces?	0.05	0.30	0.36	**0.64**
During a typical week, how often do you have fruits washed and ready for your child to eat during the day?	−0.08	0.25	0.34	**0.62**
During a typical week, how often do you have ready-to-eat fruit or vegetable snacks for your child to eat on the go or outside of your home (incl. prepared or purchased snacks)?	0.04	0.41	0.11	**0.61**
During a typical week, how often do you have your child help prepare fruits or vegetables for a meal or snack?	−0.07	0.23	0.27	**0.59**

^1^ FVPPQ = Fruit and Vegetable Parenting Practices Questionnaire; response options for each item range from never (0), sometimes (1), often (2), and almost always (3).

**Table 4:** Confirmatory Factor Analysis (CFA) Model fit and Inter-item Consistency Reliability for FVPPQ ^1^ Subscales within Racial-ethnic Groups.

FVPPQ ^1^ Subscale	Black (*n* = 106)				Hispanic White (*n* = 100)				Non-Hispanic White (*n* = 75)			
	**χ^2^ (*p*)**	**SRMR**	**CFI**	**α**	**χ^2^ (*p*)**	**SRMR**	**CFI**	**α**	**χ^2^ (*p*)**	**SRMR**	**CFI**	**α**
Pressure	19.08 (0.02)	0.05	0.98	0.82	20.30 (0.02)	0.05	0.98	0.82	35.36 (0.00)	0.06	0.97	0.86
Availability	4.77 (0.44)	0.02	1.00	0.86	2.73 (0.74)	0.02	1.00	0.65	13.13 (0.02)	0.04	0.99	0.87
Modeling	9.61 (0.09)	0.02	0.997	0.90	28.23 (0.00)	0.06	0.96	0.80	29.22 (0.00)	0.04	0.98	0.88
Child-Focused	9.72 (0.08)	0.03	0.99	0.86	4.21 (0.52)	0.03	1.00	0.77	5.00 (0.46)	0.02	1.00	0.83

^1^ FVPPQ = Fruit and Vegetable Parenting Practices Questionnaire.

**Table 5:** Confirmatory Factor Analysis (CFA): Measurement Equivalence Across Racial/Ethnic Groups or FVPPQ ^1^ Subscales.

FVPPQ Subscale	Configural vs. Metric		Metric vs. Scalar	
	Δχ^2^ (*p*) ^2^	*df*	Δχ^2^ (*p*) ^2^	*df*
Pressure	17.50 (0.06)	10	20.83 (0.53)	22
Availability	3.58 (0.89)	8	25.07 (0.12)	18
Modeling	6.62 (0.58)	8	22.27 (0.22)	18
Child-Focused	10.93 (0.21)	8	48.40 (0.00)	18

^1^ FVPPQ = Fruit and Vegetable Parenting Practices Questionnaire; ^2^ A statistically non-significant Δχ^2^ at *p* = 0.05 indicates equivalence across groups.

## Data Availability

The data presented in this study are available on request from the corresponding author. The data are not publicly available due to potential privacy and confidentiality issues. Because the sample was drawn from Head Start programs serving diverse families, public data sharing could increase the risk of participant identification, data access is therefore restricted to protect participant confidentiality.

## Supplementary Materials

The following supporting information can be downloaded at: https://www.mdpi.com/article/10.3390/nu18060974/s1, Table S1: Fruit and Vegetable Parenting Practices Questionnaire (FVPPQ): 21-Item Measure with 4 Domains of Parenting Practices. Table S2: Correlations Between Fruit and Vegetable Parenting Practice Questionnaire (FVPPQ) and Relevant Variables (*N* = 281). Refs. [20,51,52,53] are cited in Supplementary Materials.
