# Supplementary material for: Fruit and Vegetable Parenting Practices in Preschoolers: Initial Examination and Cultural Equivalency of a New Measure
Source: Nutrients. 2026 Mar 19;18(6):974. doi: 10.3390/nu18060974 (PMC13029407; doi:10.3390/nu18060974)
Supplement: Supplementary file 1 [file nutrients-18-00974-s001.zip › Table S1.pdf]

Table S1. Fruit and Vegetable Parenting Practices Questionnaire (FVPPQ): 21-Item Measure with 4 Domains of Parenting Practices

**Subscales with Individual Items**	**Original Item Source/Inspiration[1]**	**Original Dimension Label**
**Availability (5 items)**		
During a typical week, how often do you include/serve vegetables in this child's meals served at home (e.g., lunch, dinner).	"I offer vegetables without forcing my child to eat them" (Baranowski et al., 2013, p. 92).	Effective: non-directive control
During a typical week, how often do you include at least one fruit or vegetable that your child likes in meals or snacks?	"I give my child vegetables for their snacks" (Baranowski et al., 2013, p. 92)	Effective: structure
How often do you buy some fruit or vegetables when shopping for food?	"Children chose to eat foods that were served most often, and preferred what was available and acceptable in the parental household." (Nicklas et al., 2001, p. 226)	Availability
How often are vegetables in your home during a typical week (fresh, canned, jarred, frozen, or 100% vegetable juice)? Please don't think about fried vegetables like French fries.	"Children chose to eat foods that were served most often, and preferred what was available and acceptable in the parental household." (Nicklas et al., 2001, p. 226)	Availability
How often do you provide vegetables for this child in the form s/he prefers – like frozen if s/he likes frozen, raw if that is the preference, or cooked if that is preferred?	"I make vegetables easy to eat, such as cleaning, peeling, or cutting them." (Baranowski et al., 2013, p. 92)	Effective: structure
**Modeling (5 items)**		
During a typical week, how often do you eat fruit for a snack when your child can see this?	"I model healthy eating for my child by eating healthy foods myself" (Van Der Horst et al., 2017, p. 7)	Modelling
During a typical week, how often do you eat a vegetable for a snack when your child can see this?	"I model healthy eating for my child by eating healthy foods myself" (Van Der Horst et al., 2017, p. 7)	Modelling
During a typical week, how often do you eat fruit in front of this child at meals?	"I model healthy eating for my child by eating healthy foods myself" (Van Der Horst et al., 2017, p. 7)	Modelling
During a typical week, how often do you eat fruit as dessert in front of this child?	"I model healthy eating for my child by eating healthy foods myself" (Van Der Horst et al., 2017, p. 7)	Modelling
During a typical week, how often do you eat a fruit in front of your child when eating out (i.e., restaurant, fast food, buffet)?	"I model healthy eating for my child by eating healthy foods myself" (Van Der Horst et al., 2017, p. 7)	Modelling
**Child Focused (5 items)**		

During a typical week, how often do you have ready-to-eat fruit or vegetable snacks for your child to eat on the go or outside of your home (including prepared at home or purchased snacks)?	"I give my child vegetables for their snacks" (Baranowski et al., 2013, p. 92)	Effective: structure
	"Children chose to eat foods that were served most often, and preferred what was available and acceptable in the parental household." (Nicklas et al., 2001, p. 226)	Availability
During a typical week, how often do you have your child help prepare fruits or vegetables for a meal or snack?	"Encouragement of the child's involvement in meal planning and preparation" (Van Der Horst & Sleddens, 2017, p. 7)	Involvement
During a typical week, how often do you have fruits washed and ready for your child to eat during the day?	"I make vegetables easy to eat, such as cleaning, peeling, or cutting them" (Baranowski et al., 2013, p. 92)	Effective: structure
During a typical week, how often do you have vegetable pieces where your child can easily reach them?	"I make vegetables easy to eat, such as cleaning, peeling, or cutting them" (Baranowski et al., 2013, p. 92)	Effective: structure
During a typical week, how often do you have fruits ready to eat by peeling, sectioning, or cutting into small pieces?	"I make vegetables easy to eat, such as cleaning, peeling, or cutting them" (Baranowski et al., 2013, p. 92)	Effective: structure
**Pressure (6 items)**		
How often do you ask/tell this child to take a few more bites of a vegetable?	"I encourage my child to try a couple of bites of a vegetable" (Baranowski et al., 2013, p. 92)	Effective: responsiveness
	"Used to encourage consumption of foods, which was most commonly manifested as assertiveness, e.g., firmly insisting that the child ate" (Moore et al., 2007, p. 705)	Moderate pressure
During a typical week, how often to you tell/ask this child to at least eat half of their vegetables so that s/he can be done eating?	"Parent's attempts to one-sidely control the child's food intake and eating practices, through commands instructions, directives, or coercion." (Nicklas et al., 2001)	Authoritarian
	"Used to encourage consumption of foods, which was most commonly manifested as assertiveness, e.g., firmly insisting that the child ate" (Moore et al., 2007, p. 705)	Moderate pressure
During a typical week, how often you barter with this child to eat their vegetables?	"Parent's attempts to one-sidely control the child's food intake and eating practices, through commands instructions, directives, or coercion." (Nicklas et al., 2001)	Authoritarian
During a typical week, how often do you tell your child there is no dessert if they don't eat their vegetables?	"I keep my child from having sweets if they do not finish their vegetables" (Baranowski et al., 2013, p. 92)	Ineffective: control

	"Mild threats and punishments, most commonly associated with the removal of rewards used to encourage eating." (Moore et al., 2007, p. 705)	Moderate pressure
During a typical week, how often do you beg your child to eat his or her fruit or vegetables?	"Uses questions, negotiations, and reasoning in an attempt to shape or guide a child's behavior." (Nicklas et al., 2001)	Authoritative
During a typical week, how often do you warn your child you will take something away (toy, playtime) if she or he doesn't eat their fruit or vegetable.	"Parent's attempts to one-sidedly control the child's food intake and eating practices, through commands instructions, directives, or coercion." (Nicklas et al., 2001)	Authoritarian
	"I keep my child from going to play if they do not eat their vegetables" (Baranowski et al., 2013, p. 92)	Ineffective: control
	"Mild threats and punishments, most commonly associated with the removal of rewards used to encourage eating." (Moore et al., 2007, p. 705)	Moderate pressure

[1]References for the original sources/inspiration: Baranowski, T., Chen, T. A., O'Connor, T., Hughes, S., Beltran, A., Frankel, L., et al. Dimensions of vegetable parenting practices among preschoolers. *Appetite*, 2013, 69, 89–93. Moore SN, Tapper K, Murphy S. Feeding strategies used by mothers of 3–5-year-old children. *Appetite*, 2007 Nov 1;49(3):704-7; Nicklas TA, Baranowski T, Baranowski JC, Cullen K, Rittenberry L, Olvera N. Family and child-care provider influences on preschool children's fruit, juice, and vegetable consumption. *Nutrition Reviews*, 2001 Jul 1;59(7):224-35; Van der Horst K, Sleddens EF. Parenting styles, feeding styles and food-related parenting practices in relation to toddlers' eating styles: A cluster-analytic approach. PloS one. 2017 May 24;12(5):e0178149.
