# Supplementary material for: Fruit and Vegetable Parenting Practices in Preschoolers: Initial Examination and Cultural Equivalency of a New Measure
Source: Nutrients. 2026 Mar 19;18(6):974. doi: 10.3390/nu18060974 (PMC13029407; doi:10.3390/nu18060974)
Supplement: Supplementary file 1 [file nutrients-18-00974-s001.zip › Table S2.pdf]

Table S2. Correlations Between Fruit and Vegetable Parenting Practice Questionnaire (FVPPQ) and Relevant Variables (N = 281)

**FVPPQ Domain/Score**	**Child Feeding Questionnaire[1]**				**Caregivers' Feeding Style Questionnaire[2]**		**Availability[3]**		**Child FV Intake[4]**		**Parental Taste Preferences[4]**		**Child Taste Preferences[4]**		**Child BMI[5]**
	Monitoring	Restriction	Pressure	Concern	Demandingness	Responsiveness	Fruit	Veg	Fruit	Veg	Fruit	Veg	Fruit	Veg	BMI
Pressure	-.11	.15**	.18**	.00	.51***	-.37***	-.11	-.04	-.04	-.14*	-.002	-.02	-.08	-.19**	-.09
Availability	.06	.02	-.03	-.09	-.01	.27***	.14*	.27***	.20***	.27***	.15**	.20***	.07	.20***	-.10
Modelling	.06	-.01	.005	.02	.01	.26***	.31***	.34***	.29***	.33***	.17**	.18**	-.03	.17**	.04
Child Focused	.07	-.06	.05	.02	-.04	.29***	.31***	.27***	.34***	.34***	.18**	.17**	.06	.19**	-.08
Total FVPPQ	.03	.04	.08	-.01	.18**	.15*	.23***	.29***	.28***	.28***	.18**	.18**	-.001	.12*	-.08

[1]Child Feeding Questionnaire [28]; [2]Caregivers' Feeding Style Questionnaire [15]; [3]FV Availability [29]; [4]Child FV Intake and Parental and Child Taste Preferences [30,31]; [5]Child Sex-specific Body Mass Index-percentile (Epi Info, CDC, version 2007); *p < .05; ** p < .01, *** p < .001.
